# Analysis of Aneuploidy Rate and Pregnancy Outcomes in Unexplained Recurrent Pregnancy Loss Couples With Chromosome Polymorphism After PGT-A

**DOI:** 10.3389/fmed.2022.803988

**Published:** 2022-03-31

**Authors:** Mingzhu Cao, Qian Zhang, Wei Zhou, Yueting Zhu, Hongchang Li, Junhao Yan

**Affiliations:** ^1^Center for Reproductive Medicine, Cheeloo College of Medicine, Shandong University, Jinan, China; ^2^Key Laboratory of Reproductive Endocrinology of Ministry of Education, Shandong University, Jinan, China; ^3^Shandong Key Laboratory of Reproductive Medicine, Jinan, China; ^4^Shandong Provincial Clinical Research Center for Reproductive Health, Jinan, China; ^5^National Research Center for Assisted Reproductive Technology and Reproductive Genetics, Shandong University, Jinan, China

**Keywords:** chromosome polymorphism, aneuploid, recurrent pregnancy loss, preimplantation genetic testing, gender

## Abstract

**Purpose:**

The study aims to investigate whether chromosomal polymorphism affects embryo development and pregnancy outcomes of unexplained recurrent pregnancy loss (uRPL) couples undergoing PGT-A.

**Methods:**

A total of 585 couples with uRPL history who performed PGT-A were included in the retrospective study from January 2016 to December 2020. We included 415 couples with normal karyotype and 170 couples with chromosomal polymorphism. Furthermore, the polymorphism group was divided into two subgroups: 113 couples in the male group and 57 couples in the female group. The embryo development and pregnancy outcomes were analyzed in different groups.

**Results:**

The blastocyst rate and aneuploidy rate are statistically different in the normal group, male polymorphism group, and female polymorphism group. Compared with normal and female groups, the male group has a lower blastocyst rate, which is statistically different (48.3 vs. 53.9%, *p* = 0.003; 48.3 vs. 54.1%, *p* = 0.043). Moreover, the aneuploidy rate of the male polymorphism group is significantly higher than female carriers (29.5 vs. 18.6%, *p* = 0.003). However, there were no statistically significant differences in clinical pregnancy rate, early miscarriage rate, and live birth rate after PGT-A (*p* > 0.05).

**Conclusion:**

Male with chromosome polymorphism (CPM) have a lower blastocyst rate and a higher aneuploidy rate than female carriers in uRPL couples undergoing PGT-A. However, when a euploid blastocyst was first transferred, no difference in pregnancy outcomes was found between the male and female polymorphism carriers. It indicated that CPM may have an adverse effect on the embryos of male carriers with uRPL history, and the occurrence of uRPL may be decreased in male polymorphism carriers after PGT-A.

## Introduction

Chromosomal polymorphism is considered as quantitative or positional alterations in constitutive DNA heterochromatin, often occurring in the centromeric region of chromosomes 1, 9, 16, Y, and short arms of acrocentric chromosomes (ACRs), such as those in D and G groups (chromosomes 13, 14, 15, 21, and 22). Studies reported that it played an important role in spindle attachment, chromosome movement, meiotic pairing, and sister chromatid cohesion. However, the real impact of chromosomal polymorphism in the human genetics remained controversial. Some studies have shown that chromosome polymorphism (CPM) was a normal chromosome karyotype with no related phenotypic and functional effects, whereas others think that CPM may have a certain impact on infertility people and recurrent pregnancy loss (RPL) (1–4).

At present, some studies reported a close association between chromosomal polymorphisms and unexplained infertility couples with reproductive disorders. The karyotype of polymorphism accounted highly in infertilities. Cheng et al. reported that the incidence of polymorphism variants in the infertile population was higher than in fertile patients (5.53 vs. 3.74%) (3). Moreover, in other studies, the patients who have experienced RPL were found to have a higher frequency of chromosomal polymorphisms (8–15%) in comparison with patients of other infertility causes and natural pregnancy (3–10%) (4–6). Therefore, RPL may be related to CPMs based on the above studies.

There are many studies with conflicting points on whether polymorphism causes an adverse impact on the pregnancy outcomes in IVF treatments. According to the study of Hong et al., chromosomal polymorphism seemed to have no adverse effects on pregnancy outcomes of IVF-ET treatment. They found no differences between the polymorphism groups and the control group in the rates of implantation and clinical pregnancy after IVF/ICSI treatment (7). However, others reported that the rates of spontaneous miscarriage and preterm birth in infertility women with CPM were significantly higher than with normal karyotype (3).

In addition, many studies that investigated the gender factor of polymorphic carriers had reported that chromosomal polymorphism occurred more frequently in male partners than female partners within recurrent spontaneous abortion couples, which mainly involve the Y chromosome. It might lead to impaired sperm quality and quantity (4, 5, 8, 9). Ni et al. reported that the first embryo transferred rate and cumulative live birth rate of male polymorphism carriers were significantly lower than those of female carriers and normal karyotype couples in IVF/ICSI treatments (10). It may prove a connection between gender and CPMs. Therefore, we conducted further research on the embryo quality and pregnancy outcome after preimplantation genetic testing (PGT) treatment, to estimate whether chromosomal polymorphism has an impact on the outcome of ART.

Most present studies focused mainly on the analysis of IVF/ICSI reproductive outcomes in CPM carriers, instead of PGT. The embryo quality, especially the rate of aneuploid following a single oocyte retrieval, is also the most concerned issue for clinicians and patients, but never analyzed as the main outcome measure in previous chromosomal polymorphism studies. PGT can detect the quality of embryos in couples with chromosomal polymorphism, which can help us judge whether polymorphism affects pregnancy outcomes genetically. Therefore, the aim of our study is to explore whether CPM and carrier’s gender affect pregnancy outcomes, and to provide a clinical guidance about the follow-up reproductive outcomes of polymorphic couples in uRPL couples.

## Materials and Methods

### Study Population

The retrospective analysis included 585 couples who have experienced unexplained recurrent pregnancy loss (uRPL) from January 2016 to December 2020 in our center. All couples received at least one cycle of PGT treatment. Female age was from 23 to 45 years old, and basal follicle-stimulating hormone (FSH) was under 10 IU/L. Antral follicle count (AFC) in one ovary was divided into three groups: normal ovarian response (NOR) (AFC5–10), poor ovarian response (POR) (AFC < 5), and polycystic ovary (PCO) (AFC > 10), which was used to assess ovarian reserve.

The definition of RPLs is controversial. RPL is defined as two or more clinical miscarriages confirmed by ultrasound or histology and biochemical pregnancy failures, not necessarily consecutive (ASRM Practice Committee). We included couples who we considered as having uRPL history. So, we excluded pregnancy loss with clear reason who fulfilled the following criteria, to reduce other factors that interfere with embryo quality and pregnancy outcomes (11–13), including (a) abnormal chromosome karyotypes, monogenic diseases, and both male and female were diagnosed as polymorphism carriers; (b) abnormal uterine anatomy assessed by hysteroscopy, hysterosalpingography, or uterine sonography; (c) endocrine diseases, such as hyperprolactinemia, hyperthyroidism, or hypothyroidism; and (d) autoimmune factors, such as antiphospholipid syndrome. Therefore, the above is the standard of uRPL population in our data.

Then, a total of 585 uRPL couples were grouped according to the karyotype and different genders: normal group included 415 couples with normal chromosomes, the male polymorphism group consisted of 113 couples, and the female consisted of 57 couples. Ethical approval for the use and analysis of information and data from patients who underwent PGT-A was obtained from the Ethics Committee of the Center for Reproductive Medicine, Shandong University. Informed consent was obtained from all the patients included in this study.

### Study Procedure

The procedure of PGT involves performing a controlled ovarian stimulation cycle, followed by mature oocyte retrieval and ICSI with the partner’s sperm. The resulting embryos, usually at the blastocyst stage, are then biopsied. The embryo is then tested for genetic abnormalities, and only the embryos with the normal DNA are later transferred (13). Appropriate ovarian stimulation protocols were given according to the female’s age and ovarian reserve function in our center. These protocols included long and short GnRH agonist, antagonist, and others, like mild stimulation or super-long protocols. The long protocol started with GnRH agonist administrated in the mid-luteal phase of the previous cycle and combined with recombinant FSH when pituitary desensitization was achieved in this cycle. Additionally, the short protocol started with the administration of GnRH agonist and recombinant FSH together on day 2 or 3 of this cycle. The antagonist protocol was used similarly to the short protocol, but it started with a GnRH antagonist. Then, the dosage of recombinant FSH was regulated according to size of the follicle and serum E2 concentration (7). An HCG trigger for final oocyte maturation was implemented when at least two follicles with diameters ≥18 mm were detected, and oocyte retrieval was performed 34–36 h later. For all couples, fertilization was achieved by ICSI. High-quality embryos (D3) were selected according to the Gardner criteria (14). At least one morphological high-quality embryo (D3) was cultured to the blastocyst stage (D5 or D6) for trophectoderm biopsy per retrieval. A total of 2,460 blastocysts were genetically screened, of all blastocysts tested using next-generation sequencing (NGS).

The embryos of D5 or D6 were subsequently frozen after biopsy, and it was suggested that only a single euploid blastocyst can be transferred in adaptable time. Only the first embryo transfer cycle was evaluated. Mosaic embryos were not transferred in our study. The endometrium was prepared by natural ovulation cycles or other artificial cycles, depending on the individual conditions. Luteal-phase support was initiated when the endometrial thickness reached ≥7 mm and continued until 12 weeks of gestation.

The serum hCG levels were measured 14 days after embryo transferred, at which time biochemical pregnancy can be diagnosed if the hCG was ≥25 IU/L. A transvaginal ultrasound scan was performed 7th or 8th week after the embryo was transferred, and clinical pregnancy was diagnosed if an intrauterine gestational sac was observed; otherwise, it was confirmed biochemical pregnancy loss (hCG positive, no ultrasound confirmed). Pregnancy termination before gestational age of 12 weeks was considered as an early miscarriage. Live birth per retrieval was defined as the delivery of a viable infant at ≥28 weeks of gestation after the embryo was transferred.

The primary outcome is development of embryos, such as blastocyst rate and aneuploidy rate. Moreover, the miscarriage rate, clinical pregnancy rate, and live birth rate were regarded as secondary outcomes between the three groups.

### Statistical Analysis

One-way ANOVA and independent sample *t*-test were used for continuous variables. The rates and categorical variables were compared by the chi-square test and Fisher’s exact test. A value of *p* < 0.05 was considered statistically significant. A multiple linear regression model was also conducted to examine the impact of various factors on embryonic development. Chromosomal polymorphism was regarded as categorical variables were transformed to dummy variables. All the statistical analyses were performed using IBM SPSS version 25.0 software.

## Results

### The Baseline Characteristics, Embryo Quality, and Pregnancy Outcomes in Three Groups

The characteristics of normal group, male polymorphism group, and female polymorphism group (only one spouse with CPMs) are listed in Table 1. In the three groups, no statistically significant differences were observed regarding female age, basal FSH, BMI, AMH, AFC, the number of oocytes obtained and MII oocytes, ovarian stimulation protocols, and endometrial thickness on HCG day (*p* > 0.05).

**TABLE 1:** Baseline characteristics of uRPL couples.

Variable	Normal *n* = 415	Male *n* = 113	Female *n* = 57	*p*-value
Female age	34.33 ± 4.61	34.05 ± 4.94	33.58 ± 4.81	0.485
<38	70.8%(294)	72.6%(82)	71.9%(41)	0.932
≥38	29.2%(121)	27.4%(31)	28.1%(16)	
Male age	35.05 ± 4.92	34.65 ± 5.00	33.68 ± 4.77	0.130
BMI	23.83 ± 3.18	23.68 ± 3.66	23.50 ± 2.97	0.736
FSH	6.73 ± 1.84	6.58 ± 1.77	6.93 ± 2.27	0.486
AMH	3.34 ± 2.56	3.21 ± 2.05	3.55 ± 2.77	0.705
AFC				0.631
NOR (5–10)	68.7%(285)	74.3%(84)	64.9%(37)	
POR (<5)	17.6%(73)	15.0%(17)	22.8%(13)	
PCO (<10)	13.7%(57)	10.6%(12)	12.3%(7)	
Ovarian stimulation protocols				0.170*
Long	34.9%(145)	44.2%(50)	31.6%(18)	
Short	26.3%(109)	21.2%(24)	15.8%(9)	
Antagonist	33.5%(139)	31.0%(35)	47.4%(27)	
Others	5.3%(22)	3.5%(4)	5.3%(3)	
No. oocytes obtained	11.52 ± 6.12	11.67 ± 5.62	11.32 ± 6.42	0.935
No. MII oocytes	10.00 ± 5.62	10.15 ± 4.91	10.00 ± 5.82	0.967
Fertilization rate (%)	79.5%(3298/4150)	78.1%(896/1147)	81.1%(462/570)	0.348
Endometrial thickness on hCG day(cm)	0.86 ± 0.17	0.85 ± 0.15	0.90 ± 0.16	0.336

*Fisher’s exact test.

**FIGURE 1:**
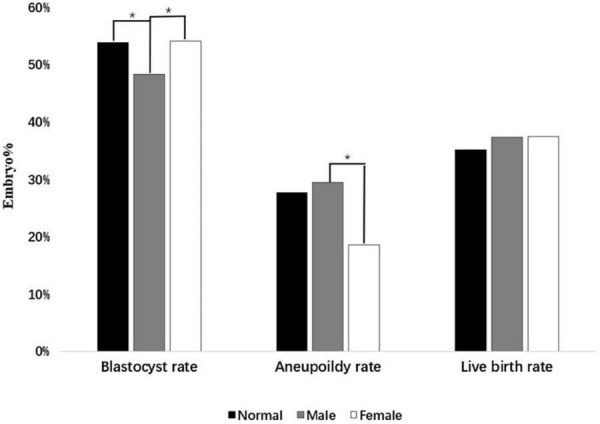
The embryo quality between three groups. The difference in Blastocyst rate, Aneuploidy rate, and Live birth rate are shown in this figure. The symbol “*” indicates a significant statistical difference (*p* < 0.05).

Moreover, Table 2 showed that the blastocyst rate and aneuploidy rate were statistically different in the normal group, male polymorphism group, and female polymorphism group. It was found that the blastocyst rate of the male polymorphism group was statistically lower than that of the normal group and female group (48.3 vs. 53.9%, *p* = 0.003; 48.3 vs. 54.1%, *p* = 0.043). More importantly, a phenomenon to higher aneuploidy rate in male polymorphism group than female carriers was noted, which was statistically significant (29.5 vs. 18.6%, *p* = 0.003). Compared with female polymorphism group, the polymorphic karyotype may have a partial effect on embryo development in men. Moreover, when analyzed the pregnancy outcomes after the first euploid embryo transplantation in three groups, the biochemical pregnancy loss rate, early miscarriage rate, clinical pregnancy rate, and live birth rate had no statistical difference (*p* > 0.05). Figure 1 also clearly showed that, male polymorphism carriers had a low blastocyst rate and a high aneuploidy rate, but there was no significant difference in pregnancy outcomes with the normal group and the female group. It probably indicated that the embryo quality of male polymorphic carriers is worse than that of female, and PGT could influence the pregnancy outcomes.

**TABLE 2:** The embryo quality and pregnancy outcomes of uRPL couples.

Variable	Normal *n* = 415	Male *n* = 113	Female *n* = 57	P1	P2	P3
**Embryo quality**						
No. D3 embryos	4.96 ± 3.35	4.83 ± 3.23	5.00 ± 3.67	NS	NS	NS
No. Blastocyst	4.28 ± 3.04	3.83 ± 2.42	4.39 ± 3.26	NS	NS	NS
Blastocyst rate (%)	53.9% (1777/3298)	48.3% (433/896)	54.1% (250/462)	0.003	0.926	0.043
Euploidy rate (%)	47.4% (711/1500)	44.3% (177/400)	51.6% (111/215)	0.262	0.246	0.080
Aneuploidy rate (%)	27.7% (416/1500)	29.5% (118/400)	18.6% (40/215)	0.485	0.005	0.003
**Pregnancy outcome**						
Biochemical pregnancy loss (%)	10.7% (23/214)	17.9% (10/56)	3.4% (1/29)	0.148	0.326*	0.089*
Clinical pregnancy(%)	67.7% (191/282)	61.3% (46/75)	70.0% (28/40)	0.297	0.773	0.355
Early miscarriage(%)	15.7% (30/191)	13.0% (6/46)	14.3% (4/28)	0.651	1.000*	1.000*
Live birth (%)	35.1% (99/282)	37.3% (28/75)	37.5% (15/40)	0.720	0.767	0.986

NS, not statistically significant; P1, normal vs. male; P2, normal vs. female; P3, male vs. female. *Fisher’s exact test.

### Prevalence of Different Types of Chromosomal Polymorphisms

**FIGURE 2:**
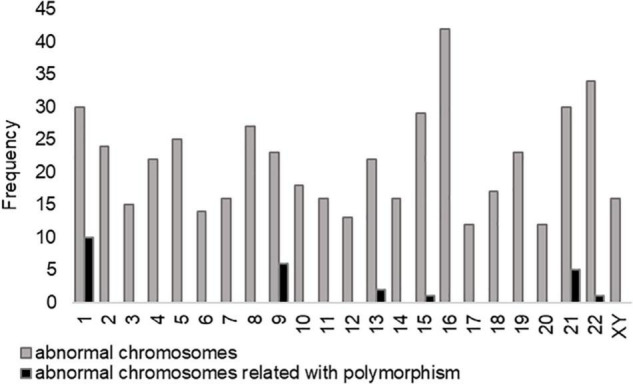
The frequency of abnormal chromosomes in the aneuploidy embryos of polymorphism group. Chromosome 16 has the highest abnormal proportion in aneuploidy embryos, followed by chromosomes 22 and 1. The proportion of abnormal chromosomes associated with chromosome polymorphism (CPM) accounts for 5.04% (25/496) in all abnormal chromosomes, and chromosomes 1, 9, and 21 appear more frequently among related chromosomes.

The incidence and types of polymorphism in 170 uRPL couples with CPM are shown in Table 3. A total of 178 chromosomal polymorphisms occurred in 170 couples, with a frequency of 119 for the male polymorphism group and 59 for female polymorphism group. Only one person in each couple was a polymorphic carrier. There were six men with ≥2 CPMs in the male polymorphism group and two women in the female polymorphism group. The type of ACR accounts for a higher proportion (30.3%) in all kinds of chromosomal polymorphism, in which chromosome 21 was the majority. The abnormality of chromosome 21 was also a common cause of miscarriage. Moreover, the most common polymorphisms observed were Y chromosome variants (31.1%) in the male group, especially Yqh+, whereas 1qh+ was the most in female group. Whether in male or female polymorphism groups, 1qh+ had a large proportion in type qh+. It indicated that the types of CPM were slightly different in different genders. In Figure 2, the abnormal chromosome 16 had the highest proportion in the aneuploidy embryos of polymorphism group, followed by chromosomes 22 and 1. Furthermore, we analyzed the influence of parental CPM in the karyotype of aneuploidy embryos (black column), to estimate whether the chromosomal polymorphisms are inherited after PGT treatments. The proportion of abnormal chromosomes associated with parental CPM accounts for 5.04% (25/496) in all abnormal chromosomes, and chromosomes 1, 9, and 21 appear more frequently among related chromosomes. It showed that the karyotype of embryos may have some correlations with parental CPM.

**TABLE 3:** Frequency of chromosomal polymorphism variation.

Types	Total	Male (*n* = 113)	Female (*n* = 57)
qh	49(27.6%)	27(22.7%)	22(37.3%)
1qh	43	23	20
9qh	3	3	0
16qh	3	1	2
ACR	54(30.3%)	30(25.2%)	24(40.7%)
13ps(s)/pstk(stk)	7	5	2
14ps(s)/pstk(stk)	7	4	3
15ps(s)/pstk(stk)	15	7	8
21ps(s)/pstk(stk)	18	10	8
22ps(s)/pstk(stk)	7	4	3
Inv(9)	38(21.3%)	25(21.0%)	13(22.0%)
Y-chromosome	37(20.8%)	37(31.1%)	–
Inv(Y)	4	4	–
Yqh–	14	14	–
Yqh+	19	19	–
Total	178	119	59

*ACR, acrocentric chromosome.*

### Multiple Linear Regression on Embryo Quality of 585 uRPL Couples

As shown in Table 4, a multiple linear regression model was conducted to comprehensively evaluate the impact of female age, female FSH, BMI, oocytes obtained, and the polymorphic carriers’ gender on the number of blastocyst and aneuploidy. It was obvious that female age, BMI, and oocytes obtained have a significant effect on blastocyst and aneuploidy (*p* < 0.05), whereas FSH does not. According to the analysis of linear regression, the number of blastocysts in the male polymorphism group was significantly lower than the normal group (β = –0.071, 95%CI –1.006 to –0.064, *p* = 0.026). It showed that male CPM may have some negative effects on embryo development.

**TABLE 4:** Multiple linear regression for the number of blastocyst and aneuploidy in uRPL couples.

Variable	No. blastocyst			No. aneuploidy		
	β	95%CI	*p*	β	95%CI	*p*
Female age	–0.162	–0.143, –0.061	<0.001	0.430	0.084, 0.121	<0.001
FSH	–0.014	–0.128, 0.083	0.675	–0.072	–0.090, 0.004	0.076
BMI	–0.078	–0.128, –0.014	0.015	–0.084	–0.054, –0.003	0.028
Oocytes obtained	0.569	0.245,0.311	<0.001	0.202	0.022, 0.052	<0.001
**Polymorphism**						
Male^a^	–0.071	–1.006, –0.064	0.026	0.021	–0.153,0.272	0.582
Female^a^	0.007	–0.563, 0.693	0.839	–0.058	–0.499, 0.065	0.131

*^a^Male group or female group compared with normal group.*

## Discussion

The previous studies have reported that the detection rate of CPM in the infertilities is higher than in the fertilities, and the overall incidence of CPM increases in infertile patients in recent years. Our study shows that the types of 1qh+, inv (9), and 21ps(s)/pstk(stk) were prevalent in uRPL people with polymorphic variations, and the incidence of chromosomal polymorphisms in men was found to be higher than that in women. The frequency of polymorphic variation on chromosome 1 is high in both male and female carriers, particularly in women (15). Moreover, chromosome 1 is also the largest one in the human chromosome group and has rich genetic material, and many genetic diseases are related to it. The abnormalities of chromosome 21 in embryos are one of the most common causes of RPL in elderly women, such as trisomy 21. The polymorphism of chromosome 9 is often thought to be associated with the meiosis of gametes. Moreover, the conclusions of some studies are slightly different from us, and they reported that chromosome 9 was the most common polymorphic variation in infertile couples, such as inv (9) and 9qh+, but not too much about the abnormalities on chromosome 1 or 22 (2, 3, 7).

Cheng et.al subdivided the female infertility group based on the reason of infertility to explore the association between CPMs and female infertility. It was found that unexplained infertility accounted for the largest proportion in CPM people than fallopian tube infertility, ovulation disorder infertility, and uterine infertility (3). Therefore, it was reasonable to suspect that unexplained infertility may be related to CPM. At the same time, the adverse pregnancy outcomes of female polymorphism carriers have also increased than normal female polymorphism carriers after excluding male polymorphism carriers. Among these pregnancy outcomes, the miscarriage rate of polymorphic female is significantly higher than that of normal karyotype female in the cause of fallopian tube infertility (6.17 vs. 1.08%). In addition, other studies have found that there was no clinical meaning when merely studying the influence of CPM on embryo quality, but the genetic effects may be presented when polymorphism coexists with other chromosomal abnormalities. For example, patients with both polymorphism and translocation (CTCPM group) have a lower rate of high-quality embryos and a higher rate of abnormal embryos than the patient with simple translocation (CT group) (*p* < 0.05). The rate of high-quality embryos was also lower in men than women in the CTCPM group (16). Therefore, we believed that the compound mutation of chromosomes polymorphism may affect the patient’s embryo quality and molecular karyotype in the above studies.

Moreover, a study reported that cooccurrences of different types of CPMs probably affect patients’ clinical phenotype, such as IVF failure, infertility, and recurrent miscarriage. It indicated that there may be an interchromosomal effect between chromosomes with polymorphism and other chromosomes (17). Others reported that chromosomal polymorphisms were associated with an increase in the occurrence risk of multinucleated embryos in the IVF/ICSI cycle. There occurs the incidence of CPM and miscarriage rate in multinucleated embryo group than that of the control group. More importantly, the phenomenon was more significant in men, but not in women (18). To sum up, clinical phenotypes may occur when CPMs and other factors work together, especially the gender factor. Similar results were presented in our study, and we found that the effect of CPM on pregnancy outcomes was related to the gender of polymorphism carriers after excluding those with complex chromosomal mutations and definite infertility reasons in our study. The embryo quality of male polymorphism carriers was worse than female carriers.

In Wilson et al.’s study, the incidence of long heterochromatic polymorphism variants in infants undergoing IVF, ICSI, and natural pregnancy was compared. It was found that infertile couples who obtained pregnancy through ART were not more likely to inherit chromosomal polymorphism than those through natural pregnancy (19). There is no significant difference in the detection rate of infant chromosomal polymorphisms, which may be because the embryos affected by CPM are naturally eliminated when embryo development or the CPMs may have no genetic effects on embryo development and pregnancy outcomes. Our study found a total of 496 abnormal chromosomes in the aneuploidy embryos of chromosome polymorphic couples, of which 25 chromosomes were associated with couples’ chromosomal polymorphism (Figure 2). The proportion accounts for 5.04% (25/496), and chromosomes 1, 9, and 21 appear more frequently among related chromosomes. It is also consistent with the occurrence of CPM as shown in Table 3. As a result, embryos are less likely to have the same chromosomal abnormalities as their parents, and the heritability of chromosomal polymorphisms is poor.

The effects of chromosomal polymorphisms on IVF/ICSI treatment are controversial as well. Some studies think that chromosomal polymorphisms had no apparent adverse pregnancy outcomes on IVF treatment. Hong et al. investigated the pregnancy outcomes after IVF treatment in male, female, and normal groups manifesting as no difference. Others reported that CPM affects the pregnancy outcomes (7). A recent study found that CPM in either male or female carriers seemed to have adverse effects on IVF/ICSI outcomes (15). Liang et al. reported that a significantly lower fertilization rate was found in infertility male compared with female and normal karyotypes (20). When stratified according to the fertilization method, the use of ICSI could increase the fertilization rate for men with chromosomal polymorphisms than IVF. Male carriers affected outcomes by decreasing the rates of fertilization, good quality embryos, clinical pregnancy, and live birth as well as increasing the biochemical pregnancy rate (*p* < 0.05), while in female carriers only by decreasing the embryo cleavage rate (*p* < 0.05). Notably, many studies included cohorts of mixed IVF or ICSI treatment. However, all cases underwent ICSI as an insemination method in our study. Moreover, a meta-analysis concluded that male chromosomal polymorphism showed lowered values for fertilization rate, cleavage rate, good quality embryos rate, and live birth rate. However, no similar correlation was found in female chromosomal polymorphism (21). The results obtained in our study could be an explanation for the results found by Ni et al. (10), it reported that male polymorphism carriers have a lower live birth rate per transfer cycle than women after IVF/ICSI treatment, and the early miscarriage rate has a rising trend (*p* < 0.05). Similar results were presented in our study, and we found there was no significant difference in pregnancy outcomes between male polymorphism carriers, female polymorphism carriers, and normal karyotype, but the aneuploidy rate of men with CPM is significantly higher than women. We speculated that this difference was mainly due to that all the transferred embryos were screened by PGT-A treatment. PGT exerted selection pressure toward the embryos to be implanted. It was probably that PGT-A could increase the euploid transferred rate and live birth rate in male polymorphism carriers compared with the couples who performed IVF/ICSI treatment. Therefore, men with CPMs in uRPL couples are more suggested to perform PGT, which decreases the risk of couples with recurrent miscarriages and reduces physical and psychological damage to women.

Morales et al. (22) revealed that CPM may have an impact on male fertility. The study analyzed the relationship between CPM and the aneuploidies in male gametes and embryos. As has been observed in the previous studies, men with CPM have an increased rate of sperm aneuploidy compared with normal men. We noted related results to Morales et al’s study. Male polymorphism carriers have a higher aneuploidy rate than female groups in uRPL couples and have generally lower rates of blastocyst and euploidy than female. The possible explanation for the above phenomenon may be the heterochromatin that plays an essential role in meiosis. Chromosomal polymorphism may impair the formation of functional gametes. Consequently, patients who are polymorphism carriers might theoretically be more susceptible to having an increased incidence of embryonic aneuploidy and impaired reproductive outcome (22). Certain biological effects of CPM could have sex-specific on cell division, particularly in the Y chromosome. In many recent studies, the variation of Y chromosome may increase the rate of errors in meiotic segregation and recombination. Some found lower fertilization rates in CPM carriers with severe oligozoospermia, compared with non-carriers with severe oligozoospermia (23). Thus, it suggested that polymorphism might have adverse effects on spermatogenesis and a negative impact on IVF outcomes.

Regarding RPL, the definition of it was the loss of two or more pregnancies before 20 weeks of gestation. It contains non-visualized pregnancy losses that combine biochemical pregnancy loss (positive hCG, no ultrasound performed) and failed PUL (positive hCG, but no pregnancy location established) (11, 12). Excluding the common causes, such as uterine abnormalities, hormonal disorders, infections, and cytogenetic abnormalities, more than 40–50% of RPL patients have no clear reason. It was considered as uRPL. So, the research to explore additional etiologies for uRPL is critically important (13, 24). Some studies reported that couples with RPL history have a significantly higher rate of sperm DNA fragmentation and a lower proportion of spermatozoa with normal morphology compared to fertile control women (8, 9). In addition, some reported that the sperm aneuploidy and DNA fragments had probably been associated with abnormal meiotic recombination. The high incidence of polymorphism in male might support the opinion that the polymorphism affects the chromosomal pairing and leads to meiotic arrest (5, 25). Therefore, sperm DNA fragmentation may be an underlying pathogenesis in uRPL people. There is a certain connection among male polymorphism variants, sperm DNA fragments, and RPL history.

Our study has some research values, but also many limitations. To our knowledge, there is currently no comprehensive investigation to report the impact of chromosomal polymorphism in uRPL couples on embryo development after PGT. Furthermore, we only included couples with uRPL after formulating the exclusion criteria, and our results may not apply to other groups of patients. The difference was significant for men and women in our study, perhaps because of the inadequacy of sample size for women. So, these results needed to be confirmed with additional studies in larger populations. Moreover, well-powered prospective studies in the number of CPMs and pregnancy outcomes are needed to fully evaluate whether polymorphism has clinical effects.

## Conclusion

The major findings of this study are that the embryo quality of male and female polymorphic groups is different in uRPL patients, male carriers with chromosomal polymorphism have a lower blastocyst rate and a higher aneuploidy rate than female carriers, but the pregnancy outcome has no difference. So, it also reminds us that the synergy of CPM and gender contribute to embryo quality. Screening the embryos may be a good option for the male polymorphic carriers with uRPL history. Preimplantation genetic testing (PGT) provides some fertility guidance for uRPL couples and reduces the occurrence of uRPL, particularly in male.

## Data Availability Statement

The raw data supporting the conclusions of this article will be made available by the authors, without undue reservation.

## Ethics Statement

Written informed consent was obtained from the individual(s) for the publication of any potentially identifiable images or data included in this article.

## Author Contributions

JY conceived and designed the study. MC analyzed the data and drafted the manuscript. QZ and WZ collected and verified the data. YZ and HL revised the manuscript. All authors contributed to conception and design of the study and were involved in interpreting the data and approved the final manuscript.

## Conflict of Interest

The authors declare that the research was conducted in the absence of any commercial or financial relationships that could be construed as a potential conflict of interest.

## Publisher’s Note

All claims expressed in this article are solely those of the authors and do not necessarily represent those of their affiliated organizations, or those of the publisher, the editors and the reviewers. Any product that may be evaluated in this article, or claim that may be made by its manufacturer, is not guaranteed or endorsed by the publisher.
